# Efficacy and safety of sacubitril/valsartan combined with dapagliflozin in non-diabetic patients with advanced chronic kidney disease

**DOI:** 10.3389/fendo.2025.1681260

**Published:** 2025-12-17

**Authors:** Huifang Wang, Xianyi Li, Chunhui Jiang, Xuemei Liu

**Affiliations:** Department of Nephrology, the Affiliated Hospital of Qingdao University, Qingdao Shandong, China

**Keywords:** chronic kidney disease, sacubitril/valsartan, dapagliflozin, combined therapy, glomerular filtration rate

## Abstract

**Background:**

Data on the effects of sacubitril/valsartan combined with dapagliflozin in non-diabetic patients with advanced chronic kidney disease (CKD) are limited. In this study, we evaluated the efficacy and safety of sacubitril/valsartan plus dapagliflozin in non-diabetic patients with advanced CKD.

**Methods:**

A single-center, prospective cohort study was conducted in non-diabetic patients with advanced CKD who had not yet initiated renal replacement therapy. Group A included 65 patients who received combined sacubitril/valsartan and dapagliflozin therapy, while Group B consisted of 59 patients treated with sacubitril/valsartan alone. Estimated glomerular filtration rate (eGFR), proteinuria, blood pressure and serum potassium levels were assessed.

**Results:**

Baseline eGFR was 36.35(31.00, 46.47) and 40.01(30.12, 45.86) mL/min/1.73m^2^ in the Group A and Group B, respectively. There was significant difference in eGFR between the two groups at month 6 [30.92(25.38, 35.38) vs. 25.42(21.58, 30.27)mL/min/1.73m^2^, *p* < 0.001]. The difference in the change in eGFR between the two groups was statistically significant (*p* < 0.001). Compared with sacubitril/valsartan alone, the combination of sacubitril/valsartan and dapagliflozin provided an additional significant reduction in blood pressure, attenuated the decline in eGFR, reduced proteinuria, and lowered the risk of hyperkalemia (*p* < 0.05).

**Conclusion:**

In non-diabetic patients suffering from advanced CKD, treatment with sacubitril/valsartan combined with dapagliflozin effectively controlled blood pressure, reduced proteinuria, slowed the progression of renal dysfunction, and did not increase the risk of adverse events, indicating a favorable safety profile.

## 1 Introduction

The prevalence of chronic kidney disease (CKD) can reach up to 17% among the general population depending on the country (1). A recent meta-analysis of 100 research studies has determined a global average prevalence of CKD at 13.4% (2). CKD is recognized as a significant risk factor for cardiovascular events and heart failure that may accelerate the progression to end stage renal disease (ESRD) compared to patients with normal kidney function (3, 4).

In the past decades, renin-angiotensin system (RAS) inhibitors have demonstrated beneficial effects in patients with CKD, slowing the progression of proteinuric CKD and reducing the levels of low-density lipoprotein cholesterol, thereby decreasing the risk of atherosclerotic vascular events (5). However, cardiac complications still lead morbidity and mortality among individuals with CKD. The PARADIGM-HF trial, a prospective comparison of neprilysin (NEP) inhibitors versus angiotensin-converting enzyme (ACE) inhibitors, demonstrated that sacubitril/valsartan significantly reduced the risk of cardiovascular death and all-cause mortality in patients with heart failure and reduced ejection fraction, and also showed a significantly lower decline in renal function, suggesting that it may have a renoprotective effect. However, patients with eGFR below 30 mL/min/1.73m^2^ have been excluded from the trial (6). Additionally, dapagliflozin, sodium-glucose co-transporter-2 (SGLT-2) inhibitor, has been proven to provide renal and cardiovascular benefits for patients with type 2 diabetes and CKD (7–9). Previous studies on sacubitril/valsartan or dapagliflozin included few patients with advanced CKD who were taking both drugs concurrently, and the efficacy and safety of sacubitril/valsartan combined with dapagliflozin in non-diabetic patients with advanced CKD remain uncertain.

Given the absence of definitive recommendations, some healthcare professionals are still hesitant to prescribe sacubitril/valsartan plus dapagliflozin for patients in advanced stages of CKD. Thus, the aim of this study is to evaluate the efficacy and safety of sacubitril/valsartan combined with dapagliflozin in non-diabetic patients with advanced CKD.

## 2 Methods

This single-center, prospective cohort study was conducted from June 2024 to December 2024 in the Department of Nephrology at the Affiliated Hospital of Qingdao University. Written informed consent was obtained from all patients before their inclusion in this prospective cohort study. The study protocol was in accordance with the provisions of the Declaration of Helsinki and was approved by the Ethics Committee of the Affiliated Hospital of Qingdao University (IRB approval number: QYFY WZLL 28698).

### 2.1 Study population

Participants were patients ≥18 years with non-diabetic CKD (eGFR 25~60 mL/min/1.73 m^2^) newly prescribed with sacubitril/valsartan who had not yet started renal replacement therapy. Patients who were receiving ACE inhibitors before initiation of sacubitril/valsartan underwent a 36-hour washout period, while patients on ARBs were transitioned directly, in accordance with guideline-based recommendations. Patients were divided into two groups according to whether they were newly initiated on dapagliflozin therapy at baseline. The exclusion criteria were as follows: (1) sacubitril/valsartan and/or dapagliflozin were discontinued or initiated during follow-up; (2) beginning renal replacement therapy; (3) treated with any sodium–glucose cotransporter 2 (SGLT2) inhibitor before enrollment; (4) no complete clinical data or incomplete follow-up; or (5) the occurrence of serious adverse events and inability to continue the study.

### 2.2 Study design and data collection

According to whether they were taking dapagliflozin, all enrolled participants were divided into the sacubitril/valsartan and dapagliflozin group (Group A) or sacubitril/valsartan group (Group B). At baseline, first dose of sacubitril/valsartan was decided by physicians according to clinical conditions. If well tolerated during follow-up, the dose would be adjusted based on each patient’s blood pressure. All therapeutic decisions in this study, including whether to initiate dapagliflozin, were made independently by treating nephrologists based on individual clinical judgment. No medication was withheld for research purposes during the study. According to the patient’s condition, patients could receive urate-lowering therapy, lipid-lowering agents among others, and the agents were allowed to be adjusted according to the condition of each patient. If a participant exited the study, their data were not included in the final analysis as they had discontinued treatment. Nevertheless, the information they provided concerning the overall rate of adverse events was still factored into the analysis. All adverse events that were deemed to be associated with the administration of sacubitril/valsartan or dapagliflozin were documented throughout the follow-up period. Baseline data recorded included sex, age, physical examination and laboratory examination were recorded in detail. Follow-up data included vital signs (blood pressure, heart rate), laboratory tests (creatinine, blood urea nitrogen, proteinuria, and potassium, etc.) and any possible side effects of the treatment or its cessation were measured at baseline, month 1, month 3, and month 6. All the data from each patient were obtained during the course of usual clinical practice at the Affiliated Hospital of Qingdao University. The eGFR was calculated by CKD-EPI equation (10).

### 2.3 Efficacy endpoints

The primary efficacy endpoint was the changes in eGFR and urinary protein levels at the end of the study in the two groups. The secondary endpoints included changes in blood pressure, serum potassium and the occurrence of any drug-related adverse events at the end of the study.

### 2.4 Statistical analysis

All statistical analyses were carried out using SPSS (SPSS Inc., Chicago, IL) version 22.0. Continuous variables were expressed as the mean ± standard deviation (SD) and compared using Student’s t-test, and non-normally distributed variables were reported as median (interquartile range [IQR]) and compared using Mann–Whitney U tests. Categorical variables were presented as numbers and percentages using Chi square test or Fisher’s exact test. Madley-Dowd et al. (11) reported that multiple imputations could reduce the bias in missing random data. We used multiple imputation methods for a small amount of missing data. A *p* value <0.05 was considered statistically significant in all analyses.

## 3 Results

### 3.1 Study cohort and their baseline characteristics

**Figure 1:**
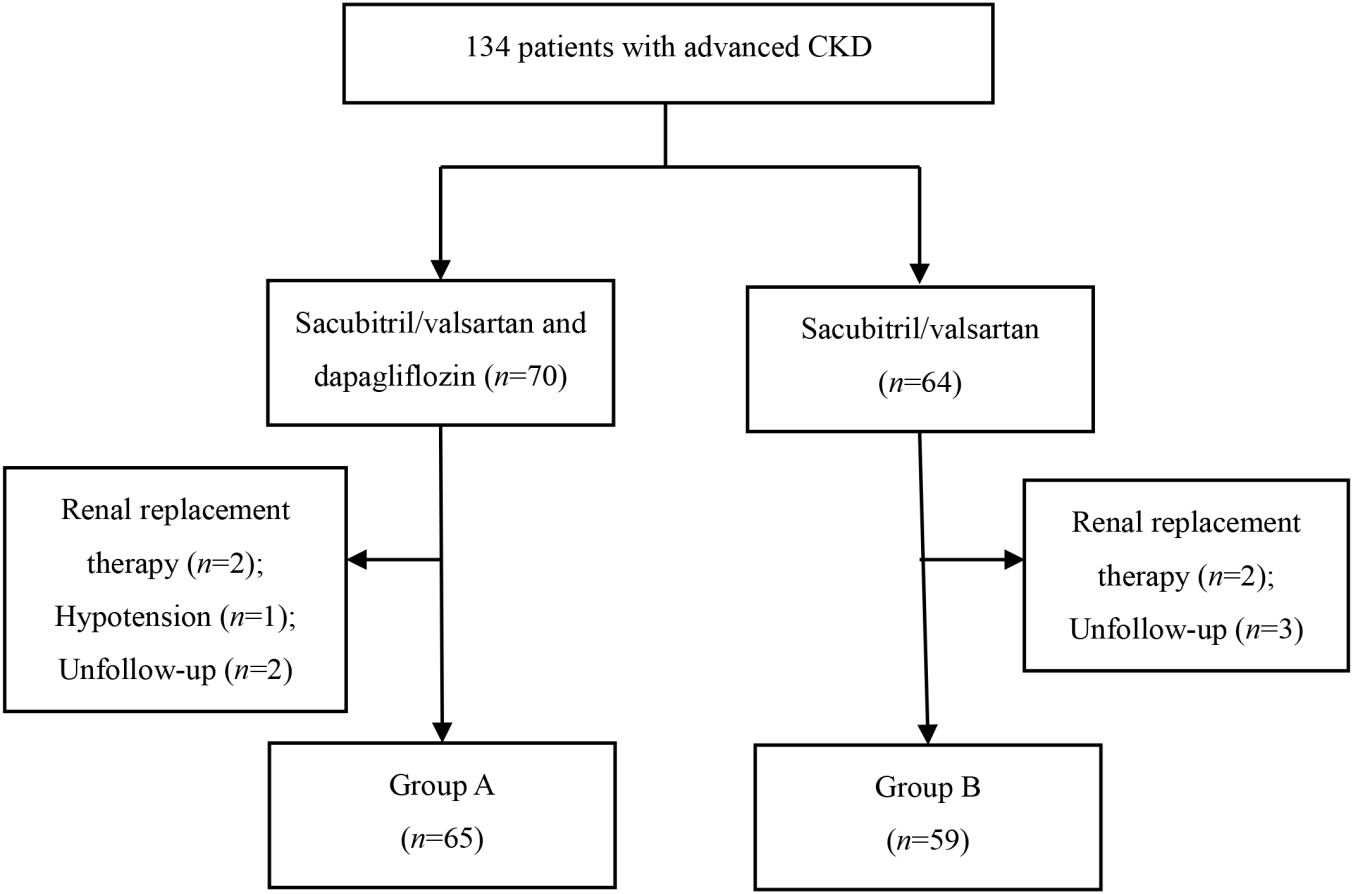
Chart of the study.

The period of follow-up was 6 months. By the end of the selection phase, there were 134 subjects recruited into our study according to the inclusion and exclusion criteria, of whom four patients required renal replacement therapy during the follow-up period and one patient discontinued the study owing to adverse events. In addition, follow-up clinical data in five patients could not be obtained owing to a change in hospital or discontinuation of attendance. Finally, For comparison between the two treatment strategies, 65 patients treated with sacubitril/valsartan combined with dapagliflozin were assigned to Group A, and another 59 patients receiving sacubitril/valsartan without dapagliflozin were assigned to Group B (**Figure 1**). All patients were from the Chinese Han population. None of the patients died during the follow-up period. Baseline characteristics of patients are listed in **Table 1**. Among the included patients, the mean age was 66.83 ± 12.49 years old and 26.6% were female. Mean eGFR was 37.70(30.99, 46.25) mL/min/1.73 m^2^ at baseline. There were no significant differences between the two groups in terms of baseline variables (*p* > 0.05) (**Table 1**).

**Table 1:** Baseline characteristics of enrolled patients.

Variables	Total (*n* = 124)	Group A (*n* = 65)	Group B (*n* = 59)	*p*
Age (years)	66.83 ± 12.49	67.69 ± 13.85	65.88 ± 10.83	0.422
Female, n (%)	33(26.6)	18(27.7)	15(25.4)	0.776
Body mass index (kg/m^2^)	25.29 ± 3.36	25.61 ± 3.34	24.94 ± 3.37	0.272
Systolic BP (mmHg)	148.92 ± 19.96	149.03 ± 21.23	148.80 ± 18.64	0.948
Diastolic BP (mmHg)	79.00(73.00, 91.00)	79.00(74.50, 90.50)	78.00(71.00, 92.00)	0.423
Cause of kidney disease				0.155
Chronic glomerulonephritis (%)	70(56.5)	40(61.5)	30(50.8)	
Hypertensive nephrosclerosis (%)	38(30.6)	16(24.6)	22(37.3)	
Tubulointerstitial disease (%)	14(11.3)	9(13.8)	5(8.5)	
Other (%)	2(1.6)	0(0.0)	2(3.4)	
Hypertension (%)	117(94.4)	60(92.3)	57(96.6)	0.300
Cardiovascular disease (%)	97(78.2)	51(78.5)	46(78.0)	0.947
Heart failure (%)	65(52.4)	33(50.8)	32(54.2)	0.699
LVEF, %	55.00(50.00, 56.00)	55.00(50.00,56.00)	55.00(46.00, 57.00)	0.607
CKD stage				0.615
CKD stage 3 (%)	97(78.2)	52(80.0)	45(76.3)	
CKD stage 4 (%)	27(21.8)	13(20.0)	14(23.7)	
Serum uric acid (μmol/L)	422.00(347.25, 465.75)	431.00(350.00, 456.00)	408.21(345.00, 494.00)	0.441
Blood urea nitrogen (mmol/L)	10.14(8.20, 13.20)	9.98(8.03, 12.19)	10.61(8.70, 14.33)	0.217
Serum creatinine (μmol/L)	148.50(130.63, 172.75)	149.00(134.40, 172.50)	143.00(128.00, 175.00)	0.797
eGFR (mL/min/1.73m^2^)	37.70(30.99, 46.25)	36.35(31.00, 46.47)	40.01(30.12, 45.86)	0.608
Serum albumin (g/L)	37.17(32.75, 40.47)	37.26(33.38, 40.95)	36.30(31.56, 40.00)	0.438
Potassium (mmol/L)	4.33 ± 0.59	4.28 ± 0.59	4.39 ± 0.58	0.294
Proteinuria (g/day)	1.69(1.24, 2.30)	1.60(1.20, 2.29)	1.80(1.30, 2.30)	0.641
Antihypertensive medication use				
β blocker (%)	62(50.0)	33(50.8)	29(49.2)	0.857
Calcium channel blockers (%)	115(92.7)	59(90.8)	56(94.9)	0.374
Diuretics (%)	26(21.0)	17(26.2)	9(15.3)	0.136
Statins (%)	69(55.6)	35(53.8)	34(57.6)	0.720
Mean dose of sacubitril/valsartan, mg/day	150(100, 200)	150.00(100.00, 200.00)	150.00(100.00, 200.00)	0.778
Mean dose of dapagliflozin, mg/day	10.0(0.0, 10.0)	10.00(10.00, 10.00)	0.00(0.00, 0.00)	<0.001

Data are presented as the mean ± standard deviation (SD), median (interquartile range) or number (percentage).

eGFR, estimated glomerular filtration rate; LVEF, left ventricular ejection fraction.

### 3.2 Comparison of eGFR and proteinuria levels between the two groups

After 6 months of follow-up, eGFR in the Group A decreased from 36.35(31.00, 46.47) to 30.92(25.38, 35.38) mL/min/1.73 m^2^; whereas, the eGFR in the Group B decreased from 40.01(30.12, 45.86) to 25.42(21.58, 30.27) mL/min/1.73 m^2^. The eGFR in both groups showed a significant decrease compared to their respective baseline values (*p* < 0.001, **Table 2**). The difference in changes of eGFR between the two groups was statistically significant (*p* < 0.001, **Table 3**).

**Table 2:** Changes in BP, eGFR, proteinuria and serum potassium levels in the two groups over the 6 month study period.

Variables	Group A (*n* = 65)	Group B (*n* = 59)	*p*
Systolic BP (mmHg)			
Baseline	149.03 ± 21.23	148.80 ± 18.64	0.948
Month 1	138.83 ± 16.49	145.10 ± 17.17	0.040
Month 3	130.03 ± 12.77	141.15 ± 16.05	<0.001
Month 6	128.83 ± 11.27	142.14 ± 15.55	<0.001
*P* ^a^	<0.001	0.002	
Diastolic BP (mmHg)			
Baseline	79.00(74.50, 90.50)	78.00(71.00, 92.00)	0.423
Month 1	79.00(71.50, 84.50)	81.00(73.00,87.00)	0.114
Month 3	75.00(68.00, 81.00)	75.00(68.00, 85.00)	0.463
Month 6	75.00(68.50, 81.00)	76.00(71.00, 85.00)	0.116
*P* ^a^	<0.001	0.050	
eGFR (mL/min/1.73m^2^)			
Baseline	36.35(31.00, 46.47)	40.01(30.12, 45.86)	0.608
Month 1	36.17(29.21, 41.91)	36.81(27.99, 43.92)	0.918
Month 3	35.53(27.73, 41.69)	32.20(26.88, 41.19)	0.533
Month 6	30.92(25.38, 35.38)	25.42(21.58, 30.27)	<0.001
*P* ^a^	<0.001	<0.001	
Proteinuria (g/day)			
Baseline	1.60(1.20, 2.29)	1.80(1.30, 2.30)	0.641
Month 1	1.50(1.05, 2.10)	1.60(1.20, 2.10)	0.494
Month 3	1.40(1.00, 2.10)	1.50(1.20, 2.00)	0.151
Month 6	1.10(0.80, 1.53)	1.50(1.20, 1.97)	<0.001
*P* ^a^	<0.001	0.036	
Potassium (mmol/L)			
Baseline	4.28 ± 0.59	4.39 ± 0.58	0.294
Month 1	4.48 ± 0.50	4.60 ± 0.78	0.303
Month 3	4.47 ± 0.52	4.54 ± 0.60	0.514
Month 6	4.46 ± 0.51	4.78 ± 0.60	0.002
*P* ^a^	0.024	<0.001	

Data are presented as the mean ± standard deviation (SD), median (interquartile range), or number (percentage).

eGFR, estimated glomerular filtration rate.

a Within-group comparison, baseline versus month 6.

**Table 3:** Comparisons of changes in clinical parameters from baseline to follow-up between group A and group B.

Variables	Group A (*n* = 65)	Group B (*n* = 59)	*P*
Blood pressure			
Mean Δ systolic BP, mmHg	-20.20 ± 18.59	-6.66 ± 16.18	<0.001
Mean Δ diastolic BP, mmHg	-7.65 ± 11.23	-3.39 ± 12.70	0.050
Laboratory values			
Mean Δ serum creatinine, μmol/L	27.04 ± 18.31	62.46 ± 27.00	<0.001
Mean Δ eGFR, mL/min/1.73m^2^	-6.82 ± 5.39	-13.77 ± 6.74	<0.001
Mean Δ Proteinuria, g/day	-0.45(-0.88, -0.20)	-0.21(-0.10, 0.20)	<0.001
Mean Δ potassium, mmol/L	0.19 ± 0.65	0.40 ± 0.65	0.075

Data are presented as the mean ± standard deviation (SD), or median (interquartile range).

Proteinuria in the Group A was 1.60(1.20, 2.29) g/day at baseline, which decreased to 1.10(0.80, 1.53) g/day at month 6 (*p* < 0.001), while in the Group B, it changed from 1.80(1.30, 2.30) g/day at baseline to 1.50(1.20, 1.97) g/day at month 6 (*p* = 0.036). There was a significant difference between the two groups in the change in proteinuria (*p* < 0.001, **Table 3**).

### 3.3 Comparison of blood pressure levels between the two groups

The systolic BP was reduced from 149.03 ± 21.23 mmHg at baseline to 128.83 ± 11.27 mmHg at the month 6 in the Group A (*p* < 0.001). The diastolic BP was reduced from 79.00(74.50, 90.50) mmHg at baseline to 75.00(68.50, 81.00) mmHg at the end of month 6 in the Group A (*p* < 0.001). The systolic BP was reduced from 148.80 ± 18.64 mmHg at baseline to 142.14 ± 15.55 mmHg at the month 6 in the Group B (*p* < 0.001). The diastolic BP was reduced from 78.00(71.00, 92.00) mmHg at baseline to 76.00(71.00, 85.00) mmHg at month 6 in the Group B (*p* = 0.050). The difference in systolic BP between the two groups was significant throughout the study period (*p* < 0.05). After 6 months of treatment, systolic blood pressure decreased significantly from baseline in Group A (-20.20 ± 18.59 mmHg) and in Group B (-6.66 ± 16.18 mmHg). The difference in changes of systolic blood pressure between the two groups was statistically significant (**Table 3**, *p* < 0.001). The diastolic blood pressure in Group A decreased from baseline (-7.65 ± 11.23 mmHg), while Group B also showed a reduction (-3.39 ± 12.70 mmHg). The difference in changes of diastolic blood pressure between the two groups was not statistically significant (**Table 3**, *p* = 0.050).

### 3.4 Safety and adverse events

The serum potassium level increased from 4.28 ± 0.59 mmol/L at baseline to 4.46 ± 0.51 mmol/L at the month 6 in the Group A (*p* = 0.024); whereas, it increased from 4.39 ± 0.58 mmol/L at baseline to 4.78 ± 0.60 mmol/L in the Group B (*p* < 0.001). At month 6, the difference in serum potassium levels between the two groups at month 6 was statistically significant (*p* = 0.002). The difference in changes of serum potassium between the two groups was not statistically significant (*p* = 0.075, **Table 3**). There was a statistically significant difference between the two groups in the number of participants who experienced hyperkalemia (11 [16.9%] vs. 22 [37.3%]; *p* = 0.010, **Table 4**).

**Table 4:** The potassium levels of the two groups at month 6.

Outcome	Group A (*n* = 65)	Group B (*n* = 59)	*P*
Potassium (mmol/L)	4.46 ± 0.51	4.78 ± 0.60	0.002
≥5.0 to <5.5 (%)	9(13.8)	17(28.8)	0.041
≥5.5 to <6.0 (%)	2(3.1)	2(3.4)	1.000
≥6.0 to <6.5 (%)	0(0)	2(3.4)	0.224
≥6.5 (%)	0(0)	1(1.7)	0.476
Total: Any potassium ≥5.0 mmol/L (%)	11(16.9)	22(37.3)	0.010

Data are presented as the mean ± standard deviation (SD), or number (percentage).

There were very few adverse events that were directly related to the study medications during the study period. Only one patient discontinued the treatment (due to hypotension) and no symptoms suggestive of orthostatic hypotension were recorded. However, two patients in the Group A and two patients in the Group B required dialysis due to a sharp decline in renal function, but there were no deaths in either group.

## 4 Discussion

To the best of our knowledge, there are limited reports on the use of sacubitril/valsartan combined with dapagliflozin for treating non-diabetic patients with advanced CKD. Our data provided evidence that, in non-diabetic patients with advanced CKD, treatment with sacubitril/valsartan combined with dapagliflozin effectively controlled blood pressure, reduced proteinuria, slowed the decline in eGFR, and did not increase the risk of adverse events.

Blood pressure control is key component in the management of CKD. Studies have indicated that among adult patients with non-dialysis dependent chronic kidney disease, over 60% have comorbid hypertension, while the rate of achieving target blood pressure control is only 30% to 65% (12). A study involving 32 patients showed that sacubitril/valsartan significantly reduced blood pressure in patients with non-dialysis dependent chronic kidney disease stages 3–4. There were no statistically significant differences in serum creatinine, eGFR, or potassium levels before and after treatment (13). Sacubitril/valsartan is a combined neprilysin inhibitor and ARB medication that extends the beneficial effects of natriuretic peptides and counteracts the adverse effects resulting from the accumulation of angiotensin II (14). Sacubitril/valsartan achieves blood pressure reduction by antagonizing the renin-angiotensin-aldosterone system (RAAS) and the sympathetic nervous system, while also increasing levels of brain natriuretic peptide, which promotes diuresis, natriuresis, and vasodilation (15). Several studies have shown that SGLT2 inhibitors can induce transient increases in urine volume and natriuresis by reducing the absorption of glucose and sodium in the proximal tubule, which is accompanied by plasma volume contraction and an increase in hematocrit, thereby contributing to a reduction in blood pressure (16). In this study, blood pressure decreased significantly in both groups. However, the reduction in systolic blood pressure was more pronounced in the combination therapy group compared to the sacubitril/valsartan group. This finding is consistent with prior research that demonstrated the complementary effects of sacubitril/valsartan and dapagliflozin on blood pressure control, likely due to their distinct mechanisms of action: sacubitril/valsartan inhibits the renin-angiotensin-aldosterone system, while dapagliflozin reduces sodium reabsorption in the proximal tubule, contributing to improved blood pressure regulation. Therefore, dapagliflozin can be used in combination with other antihypertensive agents or as a substitute for them, thereby improving the control of hypertension and providing additional benefits.

The inverse correlations between eGFR and potassium changes during treatment with sacubitril/valsartan and dapagliflozin are notable and highlight the distinct mechanisms of action of these two classes of drugs. Dapagliflozin reduces eGFR by increasing sodium delivery to the distal tubule, activating tubuloglomerular feedback, and causing afferent arteriole constriction. This lowers intraglomerular pressure, protecting kidneys. It lowers blood potassium by enhancing the electronegative charge in the tubular lumen, driving potassium secretion via principal cells in the cortical collecting duct (17). On the other hand, sacubitril/valsartan can inhibit the RAAS, which may lead to a decline in eGFR and an increase in potassium levels. The combination of sacubitril/valsartan and dapagliflozin may lead to a relatively large acute decline in eGFR in the short term. However, long-term use can reduce glomerular hyperfiltration and help maintain long-term kidney function stability, and slow the rate of long-term eGFR decline. In the PARADIGM-HF trial, the sacubitril/valsartan group showed a significantly lower incidence of renal function decline, suggesting that neprilysin inhibition may have a renoprotective effect (6). Consistent with previous findings, our study also demonstrated that, compared with sacubitril/valsartan monotherapy, the combination of sacubitril/valsartan and dapagliflozin significantly slowed the decline in eGFR (*p* < 0.05) at the 6-month follow-up. This suggests that the addition of dapagliflozin to sacubitril/valsartan provides additional renoprotective benefits, which could be attributed to the combined actions of reducing glomerular pressure, decreasing proteinuria, and improving hemodynamics. However, in our study, the combination of sacubitril/valsartan and dapagliflozin was still associated with a noticeable decline in renal function. This could be due to the fact that our participants were patients with advanced CKD, with a mean eGFR of 37.70 (30.99, 46.25) mL/min/1.73 m^2^, where a decline in renal function remains unavoidable. Overall, for non-diabetic patients with advanced chronic kidney disease (eGFR 25–60 mL/min/1.73 m^2^), combination therapy with sacubitril/valsartan and dapagliflozin can significantly delay the progression of renal dysfunction without notable adverse effects. Furthermore, the follow-up period in our study may have been too short, indicating that longer observation periods will be necessary in future studies to gain a better understanding of the impact of sacubitril/valsartan combined with dapagliflozin on renal function in CKD patients.

Proteinuria is a well-established marker of kidney damage and a predictor of adverse renal outcomes. The kidney-protective effects of SGLT2 inhibitors have previously been shown in patients with type 2 diabetes and chronic kidney disease in the CREDENCE trial (7). Its mechanism of action may involve decreasing proteinuria by promoting natriuresis and reducing intraglomerular pressure through increased sodium delivery to the distal tubule (18, 19). Previous studies have shown that both sacubitril/valsartan and SGLT2 inhibitors independently reduce proteinuria, making this combination particularly promising for advanced CKD management (9, 13). The ability of the combination therapy to significantly reduce proteinuria underscores its potential to protect against the progression of CKD. Our study also found that compared with sacubitril/valsartan monotherapy, the combination of sacubitril/valsartan and dapagliflozin demonstrated a more significant effect in reducing proteinuria and was well tolerated without notable safety concerns.

In terms of safety, both treatment regimens were generally well tolerated. However, the combination of sacubitril/valsartan and dapagliflozin led to a slight increase in serum potassium levels. While hyperkalemia was more common in sacubitril/valsartan group (37.3% vs. 16.9%, *p* = 0.010), both groups showed manageable increases in potassium. Notably, despite these changes, there were no significant adverse events related to the medications, and no deaths occurred during the study period. This is consistent with the established safety profile of sacubitril/valsartan and dapagliflozin in patients with CKD. Importantly, although hyperkalemia remains a concern, the combination of these therapies did not appear to increase the risk of severe complications when carefully monitored.

This study has several limitations. First, this study was a single-center study with a relatively small sample size. The main reason was that clinicians still had concerns about using the combination of sacubitril/valsartan and dapagliflozin in patients with mid-to-late stage CKD. Secondly, a limitation of our study is the relatively short follow-up period of six months. Although this duration was sufficient to observe significant changes in eGFR and proteinuria, a longer observation period may be necessary to fully assess the long-term effects of sacubitril/valsartan and dapagliflozin on renal function in advanced CKD patients. Future studies with extended follow-up times could provide more insight into the long-term safety and durability of these benefits. Additionally, a larger, multicenter study would help confirm the generalizability of our findings to a broader population.

## 5 Conclusion

In conclusion, our study provided evidence that the combination of sacubitril/valsartan and dapagliflozin is an effective and safe treatment for non-diabetic patients with advanced CKD. This combination therapy significantly reduces the progression of renal dysfunction, improves blood pressure control, and reduces proteinuria, with a favorable safety profile. These findings support the use of this dual therapy as a potential treatment option for advanced CKD, although further research with longer follow-up periods is needed to confirm the long-term benefits of this approach.

## Data Availability

The raw data supporting the conclusions of this article will be made available by the authors, without undue reservation.
